# Deliberative Democracy and Incompatibilities of Choice Norms

**DOI:** 10.3390/bs13120985

**Published:** 2023-11-29

**Authors:** Hannu Nurmi

**Affiliations:** Department of Philosophy, Contemporary History and Political Science, University of Turku, 20014 Turku, Finland; hnurmi@utu.fi

**Keywords:** social choice, incompatibility results, aggregation paradox, formal models of deliberation

## Abstract

Deliberative democracy aims at reaching collective decisions through mechanisms that involve flexible opinions, variable alternative sets and information gathering in the process of decision making as opposed to exogenously fixed alternative sets and preference rankings. Deliberative democracy includes elements derived from bargaining and negotiation. Among its virtues, some proponents of deliberative democracy have included the possibility that several important negative results of the theory of voting can be avoided. The basic stratagem is to dismiss the universal domain condition typically assumed in social choice results. Thus, the validity of the results escaped from is obviously not in question. The position taken in this paper is that, while in some respects plausible, the escape argument is based on a too narrow view of the incompatibility results of the social choice theory. Some fundamental paradoxes remain beyond the reach of the deliberative techniques and are even exacerbated by them. That said, the deliberative approach can certainly be adopted for making voting alternatives more meaningful to those involved.

## 1. Introduction

Over the past couple of decades, institutions resembling direct democracy have been proposed as ways of counteracting declining turnouts in Western democracies. Most proposals suggest new institutions to complement rather than replace the existing ones that are primarily of representative variety where the voters elect representatives who then make the decisions regarding legislation, policies, national budgets, etc. Under special circumstances, the decisions—e.g., major changes in the constitution, entering or leaving military or economic alliances—made or to be made by the representative institutions are subjected to a referendum. It is, however, fair to say that a vast majority of collective decisions in public bodies are made by representatives without direct recourse to voter opinions. It is quite possible then that in some issues the opinions of the representatives differ from those of their supporters so that the legislative outcomes backed by a vast majority of representatives may be supported (e.g., in opinion polls) by only a minority of voters. This possibility is sometimes called the referendum paradox (see Section 4).

Direct and representative systems resort to basically similar mechanisms in reaching outcomes, viz. voting. A different approach is pursued by the advocates of deliberative democracy. Rather than voting on a given set of decision alternatives (policy options, candidates, legislative proposals), the emphasis is on the specifics of decision alternatives, along with their formation and modification ensuing from deliberations and negotiations [1,2,3,4,5]. The deliberative institutions are occasionally advocated as devices to deal with the many negative results achieved in the social choice theory, notably Arrow’s impossibility theorem or the Gibbard–Satterthwaite theorem [6,7,8,9]. We briefly assess the success of these devices in improving democratic decision procedures. It will be argued that deliberative institutions have very limited—if any—role in escaping some quite central negative results of the social choice theory.

## 2. On Two Incompatibilities and Ways around Them

The best known and most referred to negative results in social choice theory are undoubtedly Arrows’s, Gibbard’s and Satterthwaite’s incompatibility results. They state that there are no voting rules that satisfy a set of apparently desirable properties. As will be recalled, in Arrow’s case the properties are unrestricted domain, Pareto condition, independence of irrelevant alternatives and non-dictatorship, together with the requirement that the result of applying the rule to any constellation of preference ranking of voters always results in a complete and transitive collective preference relation. In the case of the Gibbard–Satterthwaite theorem, the properties proven to be incompatible are non-dictatorship and non-manipulability, assuming that the rules are non-degenerate and universal (Formally, the rules studied by Arrow are different from those dealt with by Gibbard and Satterthwaite: the former are called social welfare functions mapping preference profiles over alternatives into collective preference relations, whereas the latter, known as social decision functions, are (singleton-valued) mappings from preference profiles over alternatives into the set of alternatives). These results are analytic truths provable from the assumptions via deductive reasoning. Their correctness is not dependent on empirical observations. Instead, it can be ascertained by studying the proofs presented in the respective sources. So, getting around or escaping from the results cannot be based on empirical observations or concocted counterexamples, but on assuming that at least one of the conditions shown to be incompatible does not hold.

The escape route from Arrow’s theorem favoured by many goes via profile restrictions. The argument goes as follows. The unrestricted domain desideratum imposed on aggregation rules is too permissive in allowing for settings that are intuitively realistic on an equal footing with those that are—again intuitively—well-nigh impossible. The incompatibility theorems require the existence of the latter types of settings. The deliberative institutions, on the other hand, exclude at least some of these settings and by so doing exclude some of the incompatibilities. Hence, through restricting the domains of voting rules, the deliberative institutions create or facilitate settings where the other conditions, except for the unrestricted domain, can be maintained.

The grounds often cited for abandoning the unrestricted domain assumption is that the profiles containing majority cycles are relatively rare in practice. More common—it is argued—are profiles where a common issue dimension prevails so that the voter preferences are single-peaked. Thus, the voters may be unanimous about an alternative that is not the worst. Under such a restriction, we can find procedures that satisfy the rest of Arrow’s assumptions. Procedures and settings that are likely to generate single-peaked preference profiles thus provide us something that could be viewed as an escape route from Arrow’s result. This is what a group of prominent deliberative democracy scholars has argued [10], and indeed, in single-peaked profile domains there is an alternative that may be regarded as a stable outcome by virtue of not being defeated by any other alternative in pairwise simple majority comparisons. The sufficiency of single-peaked preferences for guaranteeing a stable voting outcome in this sense was already established by Black [11] a few years prior to Arrow’s result. Similar results followed about a decade later [12,13,14] and, eventually, the results by Sen and Pattanaik [15] led Kramer to conclude in the early 1970s ([16], p. 286):

… thus the search for additional “similarity” conditions on individual preferences is, in a sense, over.

The main findings of List et al. suggest that deliberation—in the sense of “discussion that is substantive, balanced and civil” ([10], p. 83)—tends to change individual preferences towards single-peakedness through creating meta-agreement regarding the decision alternatives that can be represented, possibly unbeknownst to the individuals, as a policy dimension so that the preference profile has the desired similarity properties. It is not argued that the ensuing representations are single-peaked, but that the deliberative practices tend to increase the size of the subsets of voters whose preferences are single-peaked.

The empirical findings of List et al. call, however, for a few remarks. Firstly, Kramer showed decades ago that the conditions for the existence of single-peaked preferences in several dimensional policy spaces are very stringent indeed [16] (cf. however, the findings of McCubbins and Schwartz discussing special circumstances suggesting otherwise [17]). Secondly, single-peakedness is a sufficient, but not necessary, condition for the majority-rule stability of the outcomes, i.e., that they are undefeated by other alternatives by a majority of voters in pairwise contests. Indeed, it is easy to concoct examples where the Condorcet winner—that is, an alternative that defeats all the others by a majority in pairwise comparisons—is not the favourite of any (including the median) voter. Thirdly, it is well-known that in several dimensional policy-spaces the median positions of voters in each dimension (which are, by definition, the stable outcomes on the dimension) do not define an outcome in the space that would be undefeated by other alternatives. So, dimension-wise stability does not necessarily coincide with the overall stability of outcomes. These remarks notwithstanding, the deliberative theorists’ main results show that the deliberative bodies have a tendency to increase the portion of the electorate having single-peaked preferences. Thus, it can be conjectured that deliberation makes stable outcomes more likely over time. This important observation has, however, nothing to do with undermining Arrow’s theorem, but rests simply on abandoning the unrestricted domain condition that plays a crucial role in the proof of the theorem.

The other important social choice theorem discussed from the deliberative democracy angle by prominent deliberative theorists [10,18] is the one proved by Gibbard and Satterthwaite [7,9]. The theorem states the incompatibility of strategy-proofness, non-triviality, universal domain, neutrality, anonymity and non-dictatorship. It applies to social decision rules, i.e., singleton-valued mappings from preference profiles to the set of alternatives (Strategy-proofness means that under no profile is it advantageous for an individual to misrepresent his/her preferences, neutrality (anonymity) means that no alternative (individual) is discriminated for or against, non-triviality means that for any alternative there is at least one profile that leads to its election and non-dictatorship means that there is no individual whose preference dictates the voting outcome under any profile). Although no voting method used in practice is a decision rule (in the strict sense of always resulting in a single alternative), it turns out that vulnerability to strategic misrepresentation of preferences is a general feature of voting rules. List and Dryzek’s suggested way of avoiding the Gibbard–Satterthwaite theorem does not amount to abandoning any of the conditions included in the theorem, but centres on the incentives for misrepresentation of preferences. In the course of the public discussion, the participants (voters) have to justify their preferences, and in so doing they find it is to their benefit to reveal their true rather than their sophisticated preferences. So, it is argued, deliberative discussion makes preference misrepresentation unlikely because, given the effects of such behaviour in public debate (loss of trust, for example), it would not be beneficial to the individual. Hence, misrepresentations would not be of the kind alluded to in the Gibbard–Satterthwaite theorem.

Similarly, as in the case of Arrow’s theorem, the deliberative context does nothing to invalidate the theorem. Rather, it amounts to stating that one of the conditions shown to be incompatible in the theorem does not hold in the deliberative processes. The deliberative context—it is argued—induces preference changes (or restrictions) that would, ceteris paribus, make preference misrepresentation unprofitable. Thus, the deliberative context amounts to a domain restriction.

It is in the end an empirical question whether the deliberative institutions in fact make all deviations from true preferences disadvantageous for the voter. However, even supposing it does, the setting assumed hereby seems nearly utopian: not only is it assumed that the debates, arguments and counterarguments constituting the deliberation are substantive, balanced and civil, as stated above, but, perhaps more importantly, it is also assumed that the participants exert no pressure on each other, but monitor each other’s preferences objectively, are ready and willing to adjust their opinions in the course of the proceedings and are in general other-regarding (see, e.g., [19,20]). A more important counterargument to the central point of the deliberative theorists is that the process whereby the preferences are gradually revealed to the members of the deliberative body may provide incentives for misrepresenting preferences, especially under voting rules which, in the absence of this kind of information, would be hard to manipulate (For measures of vulnerability of various procedures to strategic misrepresentation of preferences, see [21]; the related important issue of safe manipulation is discussed in [22]). For example, the plurality runoff rule may not provide much impetus for insincere voting in contexts where very little or nothing is known about the other voters’ opinions, but once the preference information becomes available, incentives to deviate from sincere voting increase for some voter groups (especially those apparently having practically no chance of getting their first ranked candidates elected). So, the preference revelation in the course of deliberation may in fact prompt the voters to misrepresent their preferences, rather than discourage them from doing so.

## 3. Avoiding Collectively Irrational Outcomes

The main point of deliberative democracy is the improvement of the outcomes reached through deliberation when compared to those resulting from voting on given alternatives. Without a process-independent criterion of outcome quality, it may be difficult to assess whether one outcome is an improvement over another, except under special circumstances. An example of this is provided by the Pareto optimality criterion, which states that alternatives that are Pareto-dominated by some other alternative be excluded from collective choices. McKelvey’s results from the late 1970s showed that the pairwise majority comparison rule—a.k.a. amendment procedure—may lead to a Pareto-dominated alternative [23] (see also [24,25]). These results are derived in situations with a potentially infinite set of alternatives. A simple example involving a small set of four alternatives shows that those results also apply in finite cases. Consider the preference profile of Table 1.

Assume the following agenda of pairwise majority comparisons: 1. B vs. D (B), 2. the winner vs. A (A), 3. the winner vs. C (C). The entries in parentheses indicate the majority winner in each pairwise comparison. This procedure results in C, yet D is preferred to C by all voters. Hence, the amendment winner C is Pareto-dominated by D.

Arguably, the deliberative process would reveal that C is not a plausible choice, while the amendment procedure without general information about the voters’ preference rankings would not necessarily exclude C. This is not to say that the deliberative process would resolve the choice problem in Table 1 since the remaining A, B and D (with C deleted because of being Pareto-dominated by D) constitute a Condorcet paradox (cyclic majority) profile. However, by virtue of making the choice of a Pareto-dominated alternative unlikely, it can lead to an outcome which is preferable to that emanating from pairwise majority comparisons.

## 4. Aggregation Paradoxes

Apart from the two paradoxes discussed above, there are many others that deserve attention in evaluating the benefits of deliberative institutions. An important class consists of paradoxes that relate to aggregating outcomes reached in several electorates to yield the ‘global’ result. For example, in deciding the location of a hazardous waste storage and processing facility, there may be several mini-publics organized in a region to discuss the issue. Once these have debated and voted upon the location problem, the question is how to reach the final decision while respecting the opinions of the mini-publics. One variant of this class, sometimes called the referendum paradox, has gained some notoriety in the U.S. presidential elections [26,27]. Table 2 illustrates.

There are 15 mini-publics taking a stand on a yes–no issue. Each has 15 voters. In 10 mini-publics 2/3 of the voters vote for ‘yes’, while 1/3 vote ‘no’. The remaining five mini-publics are unanimously in favor of ‘no’. Aggregating over mini-public outcomes would thus suggest ‘yes’ as the global outcome as this is the view of 2/3 of the electorate in each of the 10 mini-publics. However, aggregating over populations indicates that 125 voters out of 225 support ‘no’. Of course, situations like this do not inevitably emerge, but their occurrence cannot be excluded a priori. either. Yet, the deliberative democracy has no instruments for dealing with such anomalies. In practice this paradox is ‘solved’ by declaring the outcome representing the opinion of the majority of mini-publics (‘yes’ in Table 2) the winner (and thus ignoring the popular vote distribution).

Much better known aggregation paradoxes bear the names of Anscombe and Ostrogorski [28,29]. The former is illustrated in Table 3 and the latter in Table 4. These paradoxes deal with dichotomous choices, here expressed as X and Y. The voters form their preferences in terms of three issue dimensions, e.g., practical competence, formal education and the language skills of candidates for a position, or environmental impact, building costs and maintenance costs for construction projects. The entries in the two tables indicate which one of the two proposals is closer to each voter’s view on each dimension (The two paradoxes are close to each other, but are not equivalent, i.e., an instance of one is not necessarily an instance of the other). Anscombe’s paradox occurs whenever there exists a majority of voters that is on the losing side on a majority of issues (In Table 3, voters 1, 2 and 3 constitute such a majority, with voter 1 being on the losing side on issues 2 and 3, voter 2 on issues 1 and 3, and voter 3 on issues 1 and 2). Ostrogorski’s paradox, in turn, occurs when (i) the outcomes resulting from aggregating individual voter opinions first over issues and then aggregating the aggregated opinions over voters and (ii) the results of aggregating first over each issue and then over all issues, suggest different overall outcomes (To wit, in Table 4 the overall opinion of voter A is X because he/she prefers X to Y on two issues out of three. The majority opinion on issue 1 is Y because three voters prefer Y to X on issue 1. The rows-first-then-column aggregation yields X, while the columns-first-then row, results in Y). Thus, the overall collective decision is ambiguous indicated by the question mark.

Both Anscombe’s and Ostrogorski’s paradoxes involve just two alternatives to choose from. Hence, they fly in the face of the common belief that the troubles of the majority rule begin when the number of alternatives is at least three. Obviously, in two-alternative settings the notion of single-peakedness loses its meaning. Therefore, the deliberative institutions cannot provide any escape routes at all to these kinds of paradoxes.

## 5. Does Deliberation Enhance Outcome Stability?

Table 1 illustrates a situation where the pairwise majority comparisons lead to a cyclic majority preference relation over the alternatives: A beats B, B beats D, D beats C (unanimously) and C beats A. The findings suggesting that deliberation tends to be associated with single-peaked preference profiles are deemed encouraging because Black showed that in these kinds of profiles the pairwise majority comparisons result not only in an unambiguous winner (the Condorcet winner), but also in a complete and transitive collective preference relation. In that sense, the outcomes can be considered stable. By definition, the Condorcet extensions satisfy outcome stability in this particular sense. However, in a more general sense the Condorcet extensions are arguably not stability-inducing at all. On the contrary, by Moulin’s result [30] Condorcet extensions exclude the possibility of always ending up with stable outcomes if stability is understood in another plausible sense, viz. as invulnerability to the no-show paradox.

The no-show paradox belongs to the class of monotonicity failures of social choice functions. In general terms, a social choice function is monotonic if an improvement of an alternative’s position in the preference relations of some individuals—everything else being fixed—is never accompanied by a deterioration of its position in the collective preference ranking ensuing from the application of the function in any profile. More specifically, consider a profile, *P*, of *n* individuals over a set, *A*, of alternatives. Let alternative *x* be among the winners (the set *C*) when function *F* is applied to profile *P* over *A*, i.e., x∈C=F(P,A). Then, *F* is monotonic if and only if $x\in F(P^{\prime })$ whenever $P^{\prime }$ is obtained from *P* by improving *x*’s position in the preference relations of one or more individuals, ceteris paribus ([31], p. 476). To show that a social choice function is non-monotonic, it is sufficient to construct a setting consisting of a preference profile over a set of alternatives where alternative *x* wins, but would not win in another profile constructed from the previous one by improving *x*’s position in some preferences with no other changes made in the profile.

Non-monotonicity reflects the lack of responsiveness in a social choice function—in fact, responsiveness is sometimes used as a synonym of monotonicity. Non-monotonic systems exhibit a form of instability in confronting the individuals with a possibility that expressing a stronger support for an alternative might turn it from a winner into a non-winner. It is clear that if, in the profile *P*, the elected alternative, *x*, is a Condorcet winner, it remains the Condorcet winner in $P^{\prime }$ as well if the latter is formed from *P*, as in the definition of monotonicity. This does not mean, however, that all Condorcet extensions are monotonic since under profiles outside the Condorcet domain (i.e., in profiles where a Condorcet winner does not exist), a change from *P* to $P^{\prime }$ may turn a winner into a non-winner under, e.g., Baldwin’s, Dodgson’s and Nanson’s rules, each of which is a Condorcet extension. The first and third one are based on the Borda count. If there are *k* alternatives, this method assigns the first-ranked alternative in an individual’s ranking k−1 points, the second-ranked one k−2 points, etc., and 0 points to the last ranked alternative. The points given to each alternative in the preference rankings are then summed up to yield the Borda score of each alternative. The collective (Borda) preference ranking is obtained as the ranking of the Borda scores; the larger the score, the higher the position. Baldwin’s method is a multi-stage one based on the Borda scores. At each stage, the alternative with the lowest Borda score is eliminated and the Borda scores are re-computed for the remaining ones. The alternative surviving all elimination stages is the Baldwin winner [32]. Using a profile with 37 individuals and three alternatives, Smith shows that Baldwin’s method is non-monotonic ([33], pp. 1036–1037) (Brandt et al. provide a simpler example to the same effect ([34], p. 541). It involves, however, dealing with ties, making the outcome dependent on the tie-breaking rule, a subject we do not deal with here). Smith’s example is reproduced in Table 5.

As usual, the profile depicts the individual preference rankings from top to bottom, with the most preferred alternatives at the top. The left side of the vertical bar in the table presents profile *P* of the above definition and the profile on the right presents profile $P^{\prime }$. The Borda scores in *P* are 40, 37 and 34 for A, B and C, respectively. Thus, C is eliminated and A ends up as the Baldwin winner. Consider $P^{\prime }$, which is obtained by improving A’s position, so that the three individuals with CBA ranking switch the positions in A’s favour to CAB. Moreover, three of the four individuals with BAC ranking move A to the top of their ranking with no other changes occurring in $P^{\prime }$ with respect to *P*. Thus, we obtain $P^{\prime }$ as a result of changes that involve improving A’ position, ceteris paribus. The Borda scores in $P^{\prime }$ are 46,31 and 34 for A, B and C, respectively. Hence, B is eliminated, whereupon C emerges as the Baldwin winner. We conclude that Baldwin’s rule is non-monotonic.

Nanson’s rule is similar to Baldwin’s, except that the elimination criterion at each stage is different from the latter’s, viz. all alternatives with at most the average Borda score are eliminated. In Table 5, the Nanson winner is A in both *P* and $P^{\prime }$. Hence the example does not demonstrate the non-monotonicity of Nanson’s rule. However, the following example does (Table 6).

Here, first D, then B and C are eliminated, whereupon A wins. Suppose now that the five left-most voters move the winner A on top of their ranking, ceteris paribus. In the resulting profile, one first eliminates B and D, after which C becomes the winner (A simpler profile in given by Brandt et al. [34], p. 540).

As a Condorcet extension, Dodgson’s method is based on the idea that a Condorcet winner should always be elected as the winner (Fishburn points out that it is not entirely fair to Dodgson to name the rule with quite a few flaws after him since he suggested several other rules and proposed counting preference switches only as a part of a complex procedure [31]. This view is shared by Tideman [35]; see also [36]. Alas, given the widespread usage, it is, however, perhaps prudent to conform with the standard usage of associating this rule to Dodgson (a.k.a. Lewis Carroll)). When one does not exist, the alternative closest to being a Condorcet winner ought to be elected. The closeness is measured by an inversion metric which, for each alternative, measures the minimum number of pairwise preference switches between adjacent alternatives required to make the alternative a Condorcet winner. The Dodgson score is thus defined for each alternative, and the one with the smallest score is the Dodgson winner. This rule has been shown to be non-monotonic by Fishburn with an example involving 19 individuals and five alternatives ([31], p. 478). It is reproduced in Table 7. Here, A is the Dodgdon winner since it requires only three preference switches to become the Condorcet winner, while the others need more switches. Suppose that a new profile is constructed from Table 7 so that the previous winner, A, is lifted to the top of the ranking of the two right-most voters, ceteris paribus. In the ensuing profile, D requires only two preference switches to become the Condorcet winner, while A still needs three such switches. Hence, Dodgson’s rule is non-monotonic.

Monotonicity is a property that is associated with electorates of a fixed size. Overall, the performance of Condorcet extensions is terms of this criterion is good, the couple of procedures just touched upon notwithstanding. Stability of outcomes can, however, be investigated in variable electorates as well, i.e., in settings where the given profile is modified either by adding or removing some groups. Moulin’s theorem deals explicitly with variable electorates ([30], p. 56). What it says, in essence, is that if there are four or more alternatives to choose from and at least 25 individuals, there is no Condorcet extension that satisfies the condition called participation (The lower bound of the number of voters has subsequently been set to 12 by Brandt et al. [37]). The theorem envisages a thought experiment whereby, starting from an outcome resulting from the application of a given rule to a profile of preference rankings, one assumes that the electorate would have been smaller due to the non-participation of a group of individuals with identical preferences. What would be the outcome in the reduced profile assuming that the active participants had maintained their preferences? If it is better for the absentees, then we have an instance of violation of the participation condition or, loosely speaking, an instance of the no-show paradox.

There are several types of participation violations, all of them undermining the stability of the social choices [38,39]. The following are of particular importance:

1. The no-show paradox (*sensu stricto* (NSP, for short)). Suppose that in an election candidate X wins. Then suppose that a group of voters all ranking Y the last (lowest) joins the electorate, ceteris paribus. If, in the enlarged electorate, Y now wins, we have an instance of NSP.
2. The more-is-less paradox (MLP). Suppose that, in an election, candidate X wins. Then suppose that a group of voters all ranking X the first joins the electorate, ceteris paribus. If now X is no longer the winner in the enlarged electorate, we have an instance of MLP.

While there is evidence suggesting that the deliberative processes can make single-peaked preference profiles more likely, they provide no escapades from participation paradoxes. As stated above, these amount to counterfactual conditional considerations of the ‘what if’ variety. These may cause the voters to downright regret their decision to cast a vote. But what about the outcomes coinciding with the Condorcet winner? Are these stable in the end? Would the retrospective thinking still give some voters a reason to regret having voted? In other words, could the voting outcome that is stable in one (Condorcet) sense be unstable in another (vulnerability to the participation paradoxes)? In a word, yes. Table 8 evaluates ten Condorcet extension methods—i.e., methods that, by definition, elect the Condorcet winner when one exists—in the light of two participation-related criteria: vulnerability to the NSP and vulnerability to the MLP. The focus here is on the Condorcet domain, that is, on the set of profiles where a Condorcet winner exists. By definition, the Condorcet extensions end up with the existing Condorcet winner in every profile in this class. So, the ensuing outcomes are stable in the Condorcet sense.

The voting rules listed in Table 8 are as follows ([40], pp. 10–13):

- The amendment rule: The candidates are subjected to pairwise comparisons according to an exogenous agenda so that, for any given pair, the candidate that gets more votes than its contestant proceeds to the contest with the next candidate in the agenda, etc., until all candidates have been present in at least one pairwise comparison. The winner of the last comparison is the overall winner. The losers in each comparison are omitted.
- Maximin rule: All pairwise comparisons of each candidate are conducted and supporting votes recorded. The candidate whose minimum support over all contests is the largest is declared the winner.
- Dodgson’s rule was discussed above.
- Nanson’s rule was discussed above.
- Baldwin’s rule was discussed above.
- Copeland’s rule: For each candidate, one determines the number of contestants he/she defeats (typically by a majority of votes) in all pairwise contests. Then, one counts the number of wins for every candidate and elects the one with the largest number of wins.
- Black’s rule: One elects the Condorcet winner if there is such a winner. Should none exist, the Borda winner is elected.
- Kemeny’s (median) rule: This rule determines the ranking that is closest to the rankings submitted by the voters in the following sense. If the number of candidates is *k*, one generates all k! strict rankings over the candidates. For each such ranking, one tallies the smallest number of individual preference switches between adjacent candidates that are required in order to make the given ranking unanimously accepted. The ranking with the smallest tally is the collective Kemeny ranking. Its first-ranked candidate is declared the winner.
- Schwartz’s rule: Determine the set of winners as the smallest set of candidates that satisfies the following condition: no candidate that is not included in the set defeats any of the candidates inside the set in pairwise comparison.
- Young’s rule: For each candidate, one defines a score that equals the smallest number of voters whose preferences have to be ignored in order to make this candidate the Condorcet winner. The candidate with the smallest score is the winner. The score ranges between 0 (when the profile contains a Condorcet winner) and n−1 (when all but one voter has to be removed to end up with a reduced profile with a Condorcet winner).

To illustrate, let us look at the NSP and Nanson’s rule in Table 9 ([41], pp. 65, 75). Here, Nanson’s rule results in B as the winner. (B is the strong Condorcet winner.) Suppose now that three new voters with the preference ranking ADBC join the electorate, ceteris paribus. In the augmented electorate there is no Condorcet winner. Computing the Borda scores results in C once A and B have first been eliminated. Thereafter, C defeats D. The Nanson winner is thus C, the lowest ranked candidate of the three added voters. Hence, Table 9 provides incentives for a significant portion of the augmented electorate to abstain.

To illustrate another ‘yes’ entry in Table 8, the vulnerability of Kemeny’s rule to NSP can be seen in Table 10, where A, the strong Condorcet winner, is at the top of the Kemeny ranking. Now suppose that, ceteris paribus, four additional voters all sharing the preference ordering BCAD join the electorate. The resulting Kemeny ranking now becomes DBCA. Hence, the lowest ranked candidate of the added voters becomes the winner, providing a rationale for the four individuals to abstain.

Table 8 shows that only two out of the ten Condorcet extensions are invulnerable to the two selected types of participation paradoxes in settings involving a Condorcet winner. In other words, the participation instability featuring in Moulin’s theorem persists, even in the restricted domain that would seem most favourable to the Condorcet extensions. The table also shows that the participation instability is related to the NSP. No rule is vulnerable to the MLP in the Condorcet domain. This follows from the fact that if *X* is the winner in a profile by virtue of being the Condorcet winner, it remains the Condorcet winner after any group of identically minded voters all ranking *X* first joins the electorate.

We have thus seen that the suggestion according to which the deliberative institutions improve the possibilities of stable outcomes implicitly refers to a specific type of stability, viz. the avoidance of majority cycles. Moulin’s theorem exhibits the incompatibility between the concept of stability in the sense of Condorcet and the stability related to incentives to participation. As it seems, the search for Condorcet stability may undermine the participation stability.

## 6. Some Formal Models of Deliberative Processes

The advocates of deliberative democracy typically present their arguments and ideas in natural languages in contradistinction to the social choice theory, which often resorts to logical and mathematical concepts. There are, however, a few works that are aimed at analysing deliberative processes via mathematical models. In this section, we take a look at some of them.

Patty and Penn present a formal model of democratic legitimacy [42]. Its basic concept is that of a principle; in order to be legitimate, a collective decision must be based on a principle. The principles are assumed to be exogenously given. They may be cyclic or acyclic. The decision is defined as legitimate just in case the process leading to it takes place in accordance with a principle. These could presumably be the subject of deliberations preceding the final collective decision. Patty and Penn prove results on the conditions of legitimate decisions. These are very general indeed. On the other hand, the results give no clues as to which principle is the one reflected in the decision reached. Since also incomplete or cyclic relations are accepted as principles, the existence theorem leaves a lot to be determined by the deliberations in order for the outcomes to be non-arbitrary.

The connection between principles and choices is also the subject of Dietrich and List’s theory [43]. The aim is to delve deeper into the background of preferences than is customary in standard choice theory by focusing on reasons or motivations for people having various kinds of preferences. Dietrich and List are not discussing deliberation as a stage of collective decision making—in fact, they do not deal with collectivities at all—but discuss ways in which preferences are formed and modified. They envisage a weighting relation over various reasons that determine a person’s preference in a decision situation. Over time, the weights may vary and consequently the preferences may vary as well. It can be envisioned that deliberative contexts are capable of bringing about such weight changes and consequently preference variations.

Perote-Peña and Piggins build a model of democracy consisting of deliberative and aggregative phases where the former precedes the latter in time [44]. In the deliberative phase, the preference profile may—under specific conditions—be modified as a result of deliberations so that opinions close to one another join to form persuasion groups. Eventually, this process may end up with a unanimous profile, effectively making the aggregation (voting) phase redundant. The group formation mechanism is driven by a parameter called the cost of persuasion. It is (inversely) related to distances between individual preference relations. An interesting finding is that the group amalgamation-based evolution makes truth-revealing processes possible by first combining groups together and then applying a specific scoring rule to the ensuing profile. Of course, this assumes that a correct or true preference relation exists. Perote-Peña and Piggins’ results are akin to those of Dryzek and List in suggesting that the deliberation phase may modify starting profiles towards such settings where a plausible outcome ensues. Perote-Peña and Piggins’ results are obtained in 3- and 4-alternative settings, which of course limits their general relevance.

An undeniable forte of deliberative democracy is the focus on reasoned choices, i.e., decisions supported by reasons, principles or arguments. These are often used interchangeably in the literature because they all seem to refer to the same thing, viz. to justifications or grounds for presenting an opinion. While the early proponents—e.g., Cohen and Habermas [45,46]—suggested that the collective decisions be results of competition between arguments where better ones wipe out worse ones so that in the end just one opinion grounded upon the best argument remains, many theorists of deliberative persuasion envisage an aggregative phase that follows the deliberation. This is the view assumed by Chung and Duggan [47]. They aim to characterize some basic institutions and practices related to deliberative democracy. Specifically, three modes of deliberation are focused upon myopic discussion, constructive discussion and debate. These are all assumed to be in line with the characteristics of the ideal deliberative procedure, *viz.*, freedom of expression, equality of participation and reciprocity often mentioned as ingredients of the democratic decision-making process. These characteristics should be viewed as ideal benchmarks of real world decision-making procedures. In particular, reciprocity, defined as the requirement that the participants support or oppose proposals using criteria that all participants accept, seems a tall order in the real world.

In myopic discussion, the decision alternatives with the arguments supporting them are subjected to a pairwise comparison with others, very much like in the amendment voting, but with the crucial difference that no vote is taken, but the winner of each comparison is determined on the basis of quality of arguments. Chung and Duggan show that, under myopic discussion, there is a non-empty limit set which can be viewed as the outcome of the discussion. Since there is nothing in the process to prevent cycles of the type ‘A is better than B on the grounds of argument $a_{1}$ and B is better that C on the grounds of argument $a_{2}$ and C is better than A on the grounds of argument $a_{3}$’, the outcome may not be a singleton. Instead, it may consist of a top cycle set. The result is very much like that of McKelvey’s [23], but differs from the latter in not including spatial information about proposals at all and defining the pairwise winning relations in terms of arguments. The upshot is that, in myopic discussions, there are no guarantees of the existence of an option that is agreed upon by all discussants other than in ‘obvious’ circumstances where a unique unassailable option *x* exists such that there is no other option that would be better than *x* by any argument at all. Clearly, this brings to mind the role of Condorcet winners in majority voting games.

Constructive discussion differs from the myopic one in making binary comparisons of decision alternatives dependent on the past history of the process. If proposal *x* is presented on the grounds of argument $a_{1}$ at stage *k* of the discussion, then proposal *y* can later be presented on the same grounds (i.e., $a_{1}$), just in case *y* is better than *x* in terms of $a_{1}$. This restriction on proposing new policy alternatives and the associated arguments guarantees, under fairly general conditions, that the discussion leads to a conclusive outcome proposal *x* such that, after *x* is reached, it remains the winning alternative in all pairwise comparisons allowed for by the defining principle of constructive discussion. Moreover, *x* is maximal in the sense that there is an argument in terms of which it is better than any other proposal. This advantage of an apparently stable outcome comes with a price: the result of constructive discussion is highly path-dependent. In fact, Chung and Duggan show (Theorem 4) that if the winner of pairwise comparisons is determined by a principle that yields a complete and transitive relation over the proposals, each proposal may the rendered the conclusive one.

By debate, Chung and Duggan refer to a setting where the discussion proceeds in accordance with an endogenous agenda in contrast to the setting of constructive discussion. Debate is modelled as a game and the focus is on equilibrium outcomes. The game is one of perfect information and the players make their proposals under the same constraints as in constructive debate. Since the aim is to find equilibria and paths leading to these, utilities are defined over positions. This is in marked contrast with the myopic and constructive discussion settings. Chung and Duggan show that, in the debate setting, an alternating sequence of reasoned proposals defines a game where a unique Nash equilibrium exists and that this equilibrium coincides with the compromise position. In 2-person games, this is a proposal, say *x*, that is ranked best by some argument and the number of arguments for which the best ranked proposal is better than *x* is less than a half of the number of arguments for both players. So, in 2-player games there is an outcome that is an equilibrium independent of the original position. Moreover, this outcome can be viewed as a kind of compromise.

The upshot of Chung and Duggan’s analysis is that, of the forms of deliberation dealt with, the debate has the best chances of leading to a satisfactory outcome regardless of the starting position. Unfortunately, the *n*-person debate setting is not explicitly covered in the article. Presumably, the analysis can be successfully extended to this more general setting in a similar way as Harsanyi ([48], pp. 196–211) extended the 2-person bargaining model to the more general setting. The other two setting types do not have such guarantees. Instead, they are likely to lead to outcomes that are path-dependent or to never ending cycles of outcomes. Of the three settings, then, two can be expected to be inconclusive and in need of being complemented by an aggregative phase in order for a democratic outcome to be reached.

## 7. Conclusions

The promise of deliberative democracy is to establish practices that are conducive to making collective choices more meaningful than the mere voting on exogenously given alternatives. The very formulation of issues to be voted upon is in the proper domain of deliberative institutions. This is definitely a different approach to voting than the one adopted in the classic social choice theory. Similarly, the idea that individual preferences are in fact expected to change in the course of deliberation differs from the classic setting. The fact that individual preferences change in the course of deliberations is by itself not necessarily democracy-enhancing, but may result from undesirable intrusions into the free expression of opinions. Hence, the ballot secrecy is an important guarantee of free expression. How to balance it with the possibility of the voters to define the issues to be decided upon is yet to be satisfactorily resolved.

In the preceding, we have approached the specific topic of deliberative escapades from the viewpoint of some important incompatibility results in social choice theory. The argument according to which the deliberative practices tend to transform the preference profiles closer to single-peakedness certainly looks promising, especially to those associating themselves with Condorcet’s intuitive concept of winning. The case for deliberation as a way of dealing with the Gibbard–Satterthwaite theory seems, however, less plausible. Rather than weakening the incentives for preference misrepresentation, the deliberation may strengthen them by disclosing preference information of other voters, which is often a necessary condition for a successful preference misrepresentation. So, there is an information trade-off in deliberative bodies: while information (often provided by experts or stake-holders) may result in more informed preferences between decision alternatives by the deliberators, the information about the opinion distributions of the participants may run counter to the efforts to find out the true preferences of the participants. The classes of paradoxes involving only two alternatives are, if anything, made more frequent by deliberative practices since they involve the crucial step of aggregating the decisions of constituent deliberative bodies.

The formal models of deliberation focus on how to lend democratic legitimacy to outcomes of collective decision making. They envisage a process consisting of deliberative and aggregative phases so that the latter are resorted to only if the former do not succeed in ending up with unique, reasoned outcomes. In the deliberative phase, the aim is to compare the quality of arguments or plausibility of reasons behind decision proposals. Deliberation is a dynamic process and under suitable conditions may lead to equilibrium outcomes in the game-theoretic sense. In myopic and constructive discussion settings, the outcomes may well require voting to end up with non-arbitrary outcomes.

The setting discussed by most authors of deliberative turn of mind is—as they willingly admit—idealistic bordering on utopian. This casts some doubt on the practical significance of the results that seem to support deliberative democracy. By opening their views for all the participants to see, a person might risk being exploited, threatened or manipulated by others later on in the deliberative process and in the eventual aggregation phase. In a similar vein, as pointed out by one reviewer of this paper, the public debate may well be biased in favour of participants with better than average rhetorical skills which are not necessarily associated with superior mastery of the substance matters.

The suggestion that deliberation as such would be prone to lead to stable outcomes sounds plausible in some contexts, but only when applied to a specific sense of stability. Similarly, the view that deliberative institutions could avoid the incompatibility results of the social choice theory is untenable: most of the results deal with exogenously given, fixed profiles and pertain to unrestricted domains, while deliberative institutions arguably involve restrictions on the domains of varying preference profiles. This does not mean that the deliberative approach to democracy is without theoretical and practical value. Its advantages over an exclusive resort to voting are obvious. If one wishes to avoid silly and/or dangerous collective decisions, one should—before voting—discuss the decision alternatives in order to exclude such implausible ones. Similarly, a deliberation might help in formulating decision alternatives in a meaningful way. Moreover, public deliberation might reveal factual information that would help the voters to form an informed opinion of the issues at hand. This, in turn, could call for information campaigns to be implemented before voting. In these campaigns, the randomly selected mini-publics can have a role in distributing information concerning the alternatives at hand. 

## Figures and Tables

**Table 1:** Amendment rule does not guarantee Pareto-optimal outcomes.

1 Voter	1 Voter	1 Voter
A	B	D
B	D	C
D	C	A
C	A	B

**Table 2:** Referendum paradox.

	Mini-Public						Row Sum
**Decision**	**1**	…	**10**	**11**	…	**15**	
yes	10	…	10	0	…	0	100
no	5	…	5	15	…	15	125

**Table 3:** Anscombe’s paradox.

Issue	Issue 1	Issue 2	Issue 3
voter 1	Y	Y	X
voter 2	X	X	X
voter 3	X	Y	Y
voter 4	Y	X	Y
voter 5	Y	X	Y

**Table 4:** Ostrogorski’s paradox.

Issue	Issue 1	Issue 2	Issue 3	Row Winner
voter A	X	X	Y	X
voter B	X	Y	X	X
voter C	Y	X	X	X
voter D	Y	Y	Y	Y
voter E	Y	Y	Y	Y
column winner	Y	Y	Y	?

**Table 5:** Baldwin’s rule is non-monotonic [33].

10	8	8	3	4	4	13	11	8	4	1
A	C	B	C	A	B	A	C	B	A	B
B	A	C	B	C	A	B	A	C	C	A
C	B	A	A	B	C	C	B	A	B	C

**Table 6:** Nanson’s rule is non-monotonic.

5	9	5	9	13	2
B	B	A	A	C	A
A	D	C	B	A	C
C	C	B	D	D	D
D	A	D	C	B	B

**Table 7:** Dodgson’s rule is non-monotonic.

5	8	4	2
A	D	B	B
B	A	E	A
C	C	D	D
D	E	A	E
E	B	C	C

**Table 8:** Ten Condorcet extensions in Condorcet domain.

Procedure	Vulnerability to NSP	Vulnerability to MLP
amendment	yes	no
maximin	no	no
Dodgson	yes	no
Nanson	yes	no
Baldwin	yes	no
Copeland	yes	no
Black	yes	no
Kemeny	yes	no
Schwartz	yes	no
Young	no	no

**Table 9:** NSP under Nanson’s rule.

5 Voters	4 Voters
B	C
C	D
D	A
A	B

**Table 10:** NSP under Kemeny’s rule.

5 Voters	3 Voters	3 Voters
D	A	A
B	D	D
C	C	B
A	B	C

## Data Availability

The data utilized in this article consist of fictitious examples devised the author for this article or by other authors as specified in the literature referred to.
